# Prognostic value of lymphocyte count in severe COVID-19 patients with corticosteroid treatment

**DOI:** 10.1038/s41392-021-00517-3

**Published:** 2021-03-02

**Authors:** Chenyang Lu, Yi Liu, Bo Chen, Hang Yang, Huifang Hu, Yi Liu, Yi Zhao

**Affiliations:** Department of Rheumatology and Immunology, West China Hospital, Sichuan University, Chengdu, China

**Keywords:** Infectious diseases, Immunotherapy

**Dear Editor**,

The outbreak of coronavirus disease 2019 (COVID-19) has so far caused over 108.2 million confirmed cases and over 2.3 million deaths all over the world as of February 14, 2021.^1^ Among in-hospital patients with COVID-19, the mortality was approximately 28%, however, the percentage increased to over 60% among critically ill patients, and over 80% among those who require mechanical ventilation.^2^ Treatment of these severe patients is becoming one of the major challenges. It has been hypothesized that a cytokine storm is the main cause of illness progression which leads to acute respiratory distress syndrome (ARDS) and organ failure. For this reason, corticosteroids and/or immunomodulatory drugs have been extensively used during the SARS-CoV-2 pandemic. However, the clinical efficacy of corticosteroids remains controversial. Moreover, corticosteroid therapy was reported to delay the clearance of SARS-CoV-2, and a high dose of corticosteroid was found to be associated with death in severe COVID-19.^3^ Therefore, to identify patients most likely benefit from corticosteroid and give precise corticosteroid therapy is essential for the management of severe COVID-19 and saving lives.

Our study included 491 consecutive patients with severe COVID-19 from the People’s Hospital of Wuhan University between February 16, 2020, and April 14, 2020. Patients were defined as severe cases when they met any of the following criteria: (1) respiratory distress, respiratory rate >30/min; (2) mean oxygen saturation ≤93% in the resting state; (3) arterial blood oxygen partial pressure (PaO_2_)/oxygen concentration (FiO_2_) ≤300 mmHg; (4) lung involvement on imaging >50% within 24–48 h. Patients who died within the first 48 h of admission were excluded because it was difficult to evaluate the effectiveness of corticosteroids. Demographic and clinical characteristics, comorbidities, laboratory findings, and chest computed tomography, and treatment details of each patient were collected and checked independently by two clinicians. The outcome was all-cause mortality during hospitalization. We conducted multivariable logistic regression analysis on factors affecting all-cause mortality of COVID-19 patients and stratified by factors including age, hypertension, C-reactive protein (CRP), d-dimer, high sensitivity cardiac troponin I (hs-cTnI), and lymphocyte count. Continuous variables were converted to categorical variables. Interaction effects were also examined between outcomes and selected variables. All statistical analyses were performed with SPSS 25.0 (IBM, Armonk, NY, USA) and R package (version 3.4.4) (Fig. 1a).

**Fig. 1:**
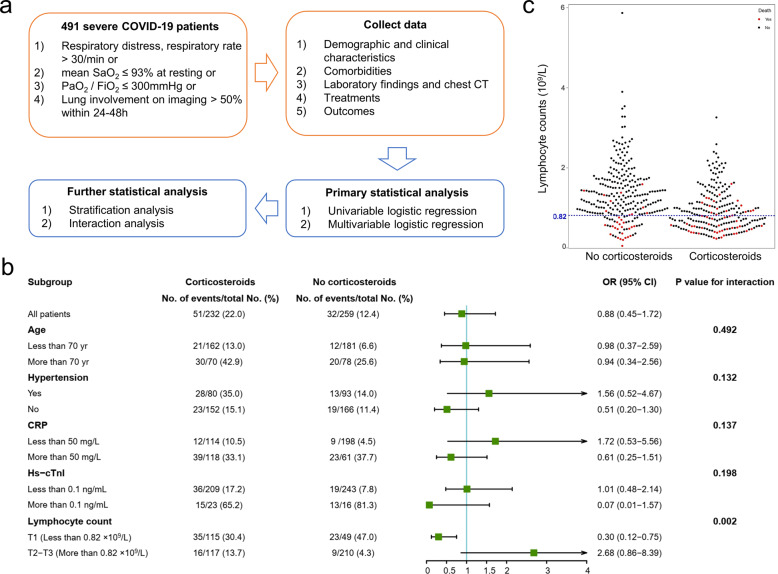
Comparison of all-cause mortality among severe COVID-19 patients receiving usual care or corticosteroid treatment. **a** The flow chart of the study. **b** Multivariable logistic regression results on mortality among severe COVID-19 patients stratified by age, blood pressure, Hs-cTnI, CRP, and lymphocyte counts. No significant heterogenicity was found between subgroups defined by age, hypertension, Hs-cTnI, and CRP. Corticosteroids use in patients with lymphocyte counts ≤0.82 × 10^9^/L was associated with a significantly reduced risk of all-cause mortality (OR, 0.30; 95% CI, 0.12–0.75), whereas no significant difference was found in patients with lymphocytes >0.82 × 10^9^/L receiving corticosteroids (OR, 2.68; 95% CI, 0.86–8.39). **c** The distribution of death in regards to lymphocyte counts showed reduced death in patients with lymphocyte counts less than 0.82 × 10^9^/L receiving corticosteroids compared to those with usual care. Red nodes indicate dead patients, black nodes indicate alive patients. hs-cTnI high sensitivity cardiac troponin I, CRP C-reactive protein, OR odds ratio, CI confidence interval

The characteristics of patients with severe COVID-19 in the cohort such as age, sex, comorbidities, clinical and laboratory parameters were shown in Supplementary Table S1. 232 (47.3%) patients received intravenous corticosteroids during hospitalization, 192 (82.8%) received 40 mg/d methylprednisolone (MP), 40 (17.2%) received 60–250 mg/d MP or equivalent doses of dexamethasone. The dosage and duration of corticosteroids used for the patients were chosen according to age, clinical symptoms, comorbidities, and lab tests. There was no significant difference in age, blood pressure, heart rate, respiratory rate, symptoms, comorbidities between the two groups (Supplementary Table S1). However, patients with corticosteroid treatment had significantly lower levels of platelet, lymphocyte, monocyte, albumin, hs-cTnI, and significantly higher neutrophil, transaminase, CRP, and D-dimer levels. In consistent with previous studies, older age, leukocytosis, cardiac injury, high levels of CRP and d-dimer, and lymphopenia were associated with the death of severe COVID-19 (Supplementary Table S2). Intriguingly, in the unadjusted model, mortality was higher in the corticosteroid group than in the usual care group, with deaths reported in 51 of 232 patients (22.0%) and in 32 of 259 patients (12.4%), respectively (OR 2.00; 95% CI 1.23–3.24; *P* = 0.005), however, the use of corticosteroids was not associated with all-cause mortality in the adjusted model (OR 0.88; 95% CI 0.45–1.72; *P* = 0.705) (Supplementary Table S2).

In order to investigate the factors influencing patients’ response to corticosteroids, we then stratified patients according to their age, blood pressure, Hs-cTnI, CRP, and lymphocyte counts. There was no significant heterogeneity of treatment effects in subgroups except that defined by lymphocyte level (Fig. 1b). Corticosteroid use in patients with lymphocyte counts ≤0.82 × 10^9^/L was associated with a significantly reduced risk of all-cause mortality (OR 0.30; 95% CI 0.12–0.75), whereas no significant difference was found in patients with lymphocytes >0.82 × 10^9^/L receiving corticosteroids (OR 2.68; 95% CI 0.86–8.39) (Fig. 1b). The distribution of death in regards to lymphocyte counts ≤0.82 × 10^9^/L also showed a reduced death rate in patients receiving corticosteroids (Fig. 1c).

Although they did not reduce deaths in the overall population, corticosteroids did reduce overall mortality of patients need ventilation. Therefore, it is important to identify such a population of patients who would benefit from corticosteroids before initiation of the therapy. In our study, the improved outcomes associated with corticosteroid in patients with lymphocytopenia is intriguing and maybe clinically important. In severe COVID-19, lymphocytes were significantly reduced, and lymphopenia was considered a potential indicator for disease severity, therapeutic response, and disease outcome. It was speculated that high levels of proinflammatory cytokines in cytokine storm syndrome (CSS) such as tumor necrosis factor (TNF)-α and interleukin (IL)-6 could induce lymphocyte deficiency. Improved outcomes with corticosteroids in this population may indicate benefit in this hyperinflammatory situation. Patients with higher lymphocyte counts were less likely to have CSS and may experience more harm than benefit when receiving corticosteroids. Furthermore, it has been suggested that early low-dose corticosteroid therapy (within 14 days from onset of symptoms, 40 mg) accelerates the recovery of CD8^+^ T cells in critical COVID-19 patients^4^ while high dose (>200 mg hydrocortisone equivalent) and early initiation (≤3 days from hospitalization) of corticosteroids were associated with a higher 28-day mortality rate and delayed viral RNA clearance.^3^ Therefore, timely intervention and dosage are also of importance when using corticosteroids.

Elevated CRP, an inflammatory marker, was observed in most patients with COVID-19, and admission CRP correlated with disease severity and predicted poor prognosis. Keller and colleagues^5^ found that glucocorticoids were associated with reduced risk of mortality or mechanical ventilation in patients with CRP ≥ 20 mg/dL, but increased risk of mortality or mechanical ventilation in patients with CRP < 10 mg/dL. However, our cohort did not find corticosteroid use was associated with lower mortality in terms of higher CRP. However, there are still several limitations in this study. Since it was a single-center study, the results may not be generalizable. As a retrospective analysis, it is subject to confounding factors and bias. We believe corticosteroids use in hospitalized patients excluded from the study reflects increased use with time because of a growing belief in their effectiveness.

Taken together, our results suggest that lymphocyte count could be a potential indicator for the identification of severe COVID-19 patients who may benefit from corticosteroid therapy. Future studies on the role of these clinical indicators in guiding corticosteroid therapy and to predict clinical response are needed.

## Supplementary information

supplement

## Data Availability

All data that support the findings of this study are available to researchers on reasonable request.
