# Supplementary material for: Prognostic value of lymphocyte count in severe COVID-19 patients with corticosteroid treatment
Source: Signal Transduct Target Ther. 2021 Mar 2;6:106. doi: 10.1038/s41392-021-00517-3 (PMC7921828; doi:10.1038/s41392-021-00517-3)
Supplement: Supplementary file 1 — supplement [file 41392_2021_517_MOESM1_ESM.docx]

Supplementary Materials for

**Prognostic value of lymphocyte count in severe COVID-19 patients with corticosteroid treatment**

Chenyang Lu, MD, PhD#, Yi Liu, MD#, Bo Chen, MD#, Hang Yang, MD, Huifang Hu, MD, Yi Liu, MD, PhD, Yi Zhao, MD *

*Correspondence to Yi Zhao, MD, Department of Rheumatology and Immunology, West China Hospital, Sichuan University, Chengdu 610041, Sichuan, China. Tel: 0086-18980605760, Fax: 0086-028-85422857. E-mail: zhao.y1977@163.com

**This PDF file includes:**

**Table S1. Baseline clinical and laboratory characteristics of study population**

|  | No corticosteroids  (n=259) | Corticosteroids  (n=232) | P-value |
| --- | --- | --- | --- |
| Baseline and demographic features | | | |
| Age | 60.90 ± 14.66 | 61.54 ± 14.93 | 0.633 |
| Female | 143 (55.21%) | 94 (40.52%) | 0.001 |
| SBP, mmHg | 132.02 ± 18.84 | 128.72 ± 18.44 | 0.051 |
| DBP, mmHg | 78.10 ± 12.21 | 77.49 ± 11.73 | 0.577 |
| Respiratory rate, breaths/min | 21.23 ± 7.81 | 21.94 ± 8.73 | 0.338 |
| Heart rate, beats/min | 87.34 ± 15.82 | 86.22 ± 17.11 | 0.453 |
| Symptoms | | | |
| Fever | 207 (79.92%) | 194 (83.62%) | 0.290 |
| Cough | 168 (64.86%) | 148 (63.79%) | 0.804 |
| Dyspnea | 99 (38.22%) | 90 (38.79%) | 0.897 |
| Fatigue | 83 (32.05%) | 80 (34.48%) | 0.567 |
| Diarrhea | 39 (15.06%) | 39 (16.81%) | 0.596 |
| Headache | 10 (3.86%) | 12 (5.17%) | 0.483 |
| Comorbidities | | | |
| Hypertension | 93 (35.91%) | 80 (34.48%) | 0.741 |
| Diabetes | 45 (17.37%) | 28 (12.07%) | 0.099 |
| Chronic lung disease | 11 (4.25%) | 15 (6.47%) | 0.273 |
| Cardiovascular disease | 29 (11.20%) | 18 (7.76%) | 0.196 |
| Malignancy | 8 (3.09%) | 8 (3.45%) | 0.823 |
| Autoimmune disease | 4 (1.54%) | 6 (2.59%) | 0.415 |
| Lab test | | | |
| RBC, ×10^12^/L | 3.98 ± 0.61 | 4.06 ± 0.64 | 0.189 |
| Hemoglobin, g/L | 122.40 ± 17.85 | 124.84 ± 17.59 | 0.128 |
| Platelet, ×10^9^/L | 234.10 ± 96.81 | 208.47 ± 83.48 | 0.002 |
| White blood cell, ×10^9^/L | 6.64 ± 5.07 | 7.10 ± 3.67 | 0.257 |
| Neutrophile, ×10^9^/L | 4.79 ± 6.25 | 5.83 ± 4.19 | 0.032 |
| Lymphocyte, ×10^9^/L | 1.36 ± 0.72 | 0.95 ± 0.52 | <0.001 |
| Monocyte, ×10^9^/L | 0.70 ± 3.21 | 0.45 ± 0.25 | 0.008 |
| Procalcitonin, ng/mL | 1.62 ± 17.37 | 0.37 ± 1.70 | <0.001 |
| ALT, U/L | 33.75 ± 33.31 | 45.35 ± 72.51 | 0.021 |
| AST, U/L | 31.80 ± 26.93 | 47.28 ± 105.53 | 0.023 |
| Albumin, g/L | 37.68 ± 4.48 | 35.93 ± 5.01 | <0.001 |
| Creatine, μmol/L | 88.32 ± 180.83 | 68.64 ± 34.85 | 0.104 |
| Hs-cTnI, ng/mL | 0.24 ± 3.12 | 0.24 ± 1.88 | <0.001 |
| CRP, mg/L | 34.59 ± 47.56 | 67.17 ± 63.17 | <0.001 |
| D-dimer, mg/L | 2.84 ± 9.83 | 7.59 ± 21.41 | 0.001 |
| Total bilirubin, μmol/L | 12.74 ± 7.40 | 15.88 ± 28.51 | 0.088 |
| Treatment | | | |
| Interferon | 29 (11.20%) | 51 (21.98%) | 0.001 |
| Arbidor | 193 (74.52%) | 177 (76.29%) | 0.649 |
| Immunoglobulin | 72 (27.80%) | 169 (72.84%) | <0.001 |
| Oseltamivir | 58 (22.39%) | 63 (27.16%) | 0.222 |

SBP, systolic blood pressure; DBP, diastolic blood pressure; RBC, red blood cell; ALT, alanine aminotransaminase; AST, aspartate aminotransferase; Hs-cTnI, high sensitivity cardiac troponin I; CRP, C-reactive protein; CI, confidence interval.

**Table S2.** **Univariable and multivariable logistic regression analysis evaluating the effect of corticosteroids and significant confounders on mortality**

|  | **Univariable analysis** | |  | **Multivariable analysis** | |
| --- | --- | --- | --- | --- | --- |
|  | **OR (95% CI)** | **P-value** |  | **OR (95% CI)** | **P-value** |
| Age (**>** 70 yr vs ≤ 70 yr) | 4.79 (2.92- 7.86) | < 0.001 |  | 3.13 (1.62- 6.04) | 0.001 |
| Diabetes (yes vs no) | 1.08 (0.56- 2.07) | 0.823 |  | - | - |
| Chronic lung disease (yes vs no) | 4.76 (2.11- 10.71) | <0.001 |  | 2.61 (0.89- 7.66) | 0.081 |
| Hypertension (yes vs no) | 2.04 (1.27- 3.29) | 0.003 |  | 1.15 (0.60- 2.18) | 0.677 |
| White blood cell, ×10^9^/L | 1.19 (1.11- 1.27) | <0.001 |  | 1.09 (1.03- 1.17) | 0.004 |
| Hemoglobin, g/L | 0.99 (0.97- 1.00) | 0.072 |  | - | - |
| Platelet (> 300×10^9^/L vs ≤ 300×10^9^/L) | 0.99 (0.99-1.00) | <0.001 |  | 0.46 (0.18- 1.18) | 0.107 |
| Monocyte, ×10^9^/L | 0.53 (0.19- 1.46) | 0.22 |  | - | - |
| Lymphocyte (T2-T3 vs T1) | 0.16 (0.09, 0.28) | <0.001 |  | 0.36 (0.18- 0.71) | 0.003 |
| CRP (> 50 mg/L vs ≤ 50 mg/L) | 7.34 (4.28- 12.59) | <0.001 |  | 2.97 (1.51- 5.85) | 0.002 |
| D-dimer (> 1mg/L vs ≤ 1mg/L） | 6.08 (3.44- 10.74) | <0.001 |  | 2.26 (1.10- 4.65) | 0.027 |
| ALT (> 40 U/L vs ≤ 40U/L) | 1.24 (0.74- 2.06) | 0.419 |  | - | - |
| AST (> 40 U/L vs ≤ 40U/L) | 3.22 (1.97- 5.27) | <0.001 |  | 1.86 (0.95- 3.62) | 0.070 |
| Creatinine(>111μmol/L vs ≤111μmol/L) | 7.79 (3.70- 16.43) | <0.001 |  | 2.01 (0.71- 5.70) | 0.191 |
| Procalcitonin, ng/mL | 1.01 (0.99- 1.02) | 0.393 |  | - | - |
| Hs-cTnI (> 0.1 ng/mL vs ≤ 0.1 ng/mL) | 18.37 (8.66- 38.99) | <0.001 |  | 2.99 (1.18- 7.54) | 0.021 |
| Corticosteroid (yes vs no) | 2.00 (1.23- 3.24) | 0.005 |  | 0.88 (0.45- 1.72) | 0.705 |

T1, tertile 1, **≤** 0.82×10^9^/L; T2-T3, tertile 2-tertile 3, > 0.82×10^9^/L; CRP, C-reactive protein; ALT, alanine aminotransaminase; AST, aspartate aminotransferase; Hs-cTnI, high sensitivity cardiac troponin I; OR, odds ratio; CI, confidence interval.
